# OTULIN is a new target of EA treatment in the alleviation of brain injury and glial cell activation via suppression of the NF-κB signalling pathway in acute ischaemic stroke rats

**DOI:** 10.1186/s10020-021-00297-0

**Published:** 2021-04-09

**Authors:** Hongbei Xu, You Wang, Yong Luo

**Affiliations:** 1 Department of Neurology, The Affiliated Hospital of Guizhou Medical University, Guizhou, 550004 China; 2 Department of Neurology, The First Affiliated Hospital of Chongqing Medical University, Chongqing, 400016 China; 3 Laboratory Research Center, The First Affiliated Hospital of Chongqing Medical University, Chongqing, 400016 China; 4 Department of Neurology, The Second Affiliated Hospital of Chongqing Medical University, Chongqing, 400016 China

**Keywords:** Acute ischaemic stroke, Electroacupuncture, OTULIN, NF-κB signalling pathway, Brain injury, Glial activation

## Abstract

**Objective:**

Ovarian tumour domain deubiquitinase with linear linkage specificity (OTULIN) is a potent negative regulator of the nuclear factor-κB (NF-κB) signalling pathway, and it plays a strong neuroprotective role following acute ischemic stroke. Electroacupuncture (EA) is an effective adjuvant treatment for reducing brain injury and neuroinflammation via the inhibition of NF-κB p65 nuclear translocation, but the underlying mechanism is not clear. The present study investigated whether OTULIN was necessary for EA to mitigate brain injury and glial cell activation in a transient middle cerebral artery occlusion (tMCAO) model in rats.

**Methods:**

An acute ischaemic stroke model was established via tMCAO surgery in Sprague–Dawley (SD) rats. EA was performed once daily at “Baihui (GV 20)”, “Hegu (LI 4)”, and “Taichong (LR 3)” acupoints. The effect of EA on the spatiotemporal expression of OTULIN in the ischaemic penumbra of the cerebral cortex was detected within 7 days after reperfusion. The effects of OTULIN gene silencing on EA neurological deficits, cerebral infarct volume, neuronal damage, the activation of microglia and astrocytes, the contents of tumour necrosis factor alpha (TNF-α), interleukin-1 beta (IL-1β) and interleukin-6 (IL-6), and the expression of p-IκBa, IκBa and nucleus/cytoplasm NF-κB p65 protein were assessed.

**Results:**

EA treatment increased endogenous OTULIN expression, which peaked at 48 h. Enhanced OTULIN was primarily located in neurons, but a small amount of OTULIN was detected in microglia. OTULIN silencing obviously reversed EA neuroprotection, which was demonstrated by worsened neurobehavioural performance, cerebral infarct volume and neuronal injury. The inhibitory effect of EA on the NF-κB pathway was also attenuated by enhanced IκBα phosphorylation and NF-κB p65 nuclear translocation. EA partially inhibited the transformation of microglia and astrocytes from resting states to activated states and reduced the secretion of TNF-α, IL-1β and IL-6. However, these preventive effects were reversed after the silencing of OTULIN expression.

**Conclusions:**

OTULIN provides a new potential therapeutic target for EA to alleviate acute ischaemic stroke-induced brain injury and the activation of glial cells, which are related to suppression of the NF-κB signalling pathway.

## Introduction

Ischaemic stroke-induced cell injury initiates the release of danger signals that activate the immune system in the central nervous system (CNS) (Corps et al. 2015). The subsequent sterile inflammatory response primarily involves the innate immune system, with the activation of resident immune cells of the central nervous system (CNS) and a rapid infiltration of peripheral immune cells (Li et al. 2020; Lin et al. 2020; Shan et al. 2020; Wang et al. 2020). The nuclear factor-kappa B (NF-κB) signalling pathway is considered a “master regulator” because it dominates the initiation and development of cerebral ischaemia-induced brain injury (Kaushal and Schlichter 2008; Liu et al. 2017). The p65/p50 subunit dimer is the main form of NF-κB, and it is primarily located in the cytoplasm where it combines with NF-κB inhibitor protein α (IκBα) in a resting state. Following activation by upstream kinases after ischaemia onset, IκBα is degraded and phosphorylated, which leads to the nuclear translocation of the p65 and binding to NF-κB-responsive genes (Mattson and Camandola 2001). Inhibition of NF-κB activation is an effective strategy for reducing acute ischaemic stroke-induced brain injury and inflammation (Li et al. 2019; Liang et al. 2019; Tan et al. 2019).

Recombinant tissue plasminogen activator (r-tPA) remains the only FDA-approved pharmacological treatment for stroke patients. However, few patients benefit from it because of the rigid therapeutic time window and severe side effects. Therefore, complementary and/or alternative medicines are urgently needed to treat ischaemic stroke worldwide. Electroacupuncture (EA) is a general complementary therapy for post-stroke patients in China because it is more readily controlled, standardized, and objectively measurable (Cai et al. 2017; Chen et al. 2020; Fan et al. 2020; Zhang et al. 2020; Zhu et al. 2020). Consistent with other reports (Liu et al. 2016), we demonstrated that the application of EA during the acute phase of cerebral ischaemic injury exerted potent neuroprotective effects by suppressing overactivation of the NF-κB signalling pathway (Zhan et al. 2016; Jiang et al. 2017; Xu et al. 2018a, b). However, the specific mechanism was not clear, and further investigation is needed.

Ovarian tumour domain deubiquitinase with linear linkage specificity (OTULIN) is a newly discovered deubiquitinase that selectively hydrolyses linear ubiquitin chains assembled by the linear ubiquitin chain assembly complex (LUBAC), and it is an essential endogenous negative regulator of inflammatory responses (Elliott et al. 2014; Damgaard et al. 2016, 2019; Zhou et al. 2016; Nabavi et al. 2019). OTULIN is expressed in immune cells, such as T cells, B cells, and natural killer cells and prominently expressed in dendritic cells and macrophages, and it is critical in the NF-κΒ-dependent inflammatory signalling pathway (Damgaard et al. 2016). OTULIN protected the liver against cell death, inflammation, fibrosis, and cancer (Damgaard et al. 2020; Verboom et al. 2020), and it is involved in the regulation of autophagy initiation and autophagosomes (Chu et al. 2020). However, the role of OTULIN in the CNS is poorly understood, and its role in cerebral ischaemia is limited to our previously published study (Xu et al. 2018a, b). OTULIN was enriched in activated microglia, and OTULIN overexpression ameliorated microglia-mediated neuroinflammation by regulating NF-κB activation in focal cerebral ischaemia/reperfusion rats (Xu et al. 2018a, b).

Our previous studies demonstrated that neuronal zinc finger protein A20 and cylindromatosis (CYLD), which are important deubiquitinases that negatively regulate the NF-κB pathway, were required for EA to alleviate the excessive inflammatory reaction in acute ischaemic rats (Zhan et al. 2016; Jiang et al. 2017). The deubiquitination activity of OTULIN is more critical than A20 and CYLD in regulating the NF-κB pathway (Keusekotten et al. 2013; Aksentijevich and Zhou 2017). Therefore, we hypothesised that EA would also alleviate brain injury, glial activation and neuroinflammation following cerebral ischaemia by regulating OTULIN. The present study characterized the spatiotemporal expression of OTULIN, the effect of EA on OTULIN expression, and the possible mechanism of EA regulation of OTULIN-mediated neuroprotection.

## Materials and methods

### Animals

A total of 313 Sprague–Dawley (SD) rats (250–300 g) were purchased from the Experimental Animal Center of Chongqing Medical University. All rats were housed under a 12/12-h light and dark cycle and allowed free access to food and water.

### Establishment of the transient middle cerebral artery occlusion (tMCAO) model

The model was performed according to a previously described method (Longa et al. 1989; Qin et al. 2013). Briefly, rats were initially anaesthetized with 3.5% chloral hydrate (1 ml/100 g, intraperitoneally). After a midline neck incision, the bifurcation of the right common carotid artery (CCA) was exposed, and a heparin-dampened nylon monofilament with a rounded tip was advanced to block the origin of the right middle cerebral artery (MCA). Two hours after MCA occlusion, rats were re-anaesthetized, and the monofilament was gently withdrawn to restore blood flow. The same surgical procedures, except the insertion of a monofilament to the origin of the MCA, were performed in the Sham group. Successful tMCAO and reperfusion was determined by a decrease in the regional cerebral blood flow to 20% and recovery to > 80% of the baseline as monitored using a laser-Doppler flowmeter (PeriFlux 5000, Perimed AB, Sweden). Throughout the surgery, the body temperature was continuously maintained at 38 ± 0.5 °C using a thermostatically controlled infrared lamp (FHC, Bowdoinham, ME, USA). After rats recovered from anaesthesia, the Longa score (Longa et al. 1989) was used to assess neurological deficits. Rats scoring 2 and 3 were included, as previously described (Xu et al. 2018a, b).

### Electroacupuncture treatment

**Fig. 1:**
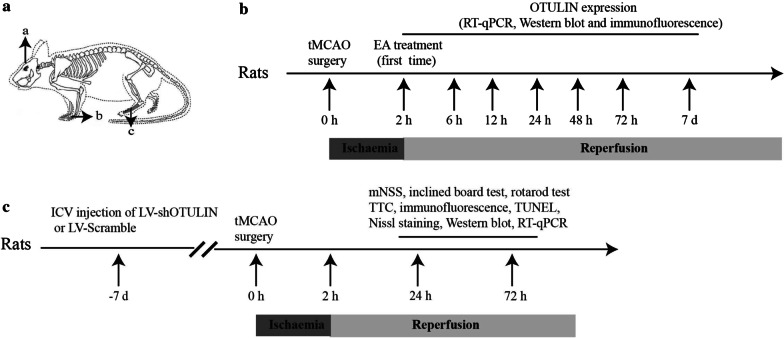
The experimental timeline. **a** EA was performed at the indicated acupoints, a: Baihui acupoint (GV 20); b: Hegu acupoint (LI 4) (the figure comes from PMID 27453543, and permission for use was obtained from the author.); c: Taichong acupoint (LR 3). **b** The characteristics of OTULIN expression were detected at 6 h, 12 h, 24 h, 48 h, 72 h, and 7 d after reperfusion. **c** The role of OTULIN in the neuroprotective, neuroinflammatory and neuronal injury effects of EA were examined

EA treatment was performed as described previously (Qin et al. 2013; Xie et al. 2016). The acupoints “Baihui (GV 20)”, “Hegu (LI 4)”, and “Taichong (LR 3)” on the left side of the rats were chosen (as shown in Fig. 1a). “Baihui (GV 20)” is located at the intersection of the sagittal midline and a line connecting the two ears. “Hegu (LI 4)” is at the second metacarpal midpoint of the radial side, and “Taichong (LR 3)” is at the second toe tibial collateral at the rear of the phalanx. One electrode in one pair of EA treatment was connected to the EA needle on the "Baihui" acupoint, and the other electrode in the same pair that formed a loop with the "Baihui" acupoint was wrapped in wet gauze and fixed on the animal's right hind limb. The "Hegu" and "Taichong" acupoints were connected to the two electrodes in another pair of EA treatment. The needles were inserted into each acupoint at an angle of 15°–45° with a 1-mm depth at "Baihui" and 2-mm depth at "Hegu" and "Taichong". The needles were connected to the EA instrument (Model no. 227033; Beijing Jinggong Ltd., China). The acupoints were stimulated with an intensity of 1 mA and a frequency of 2/20 Hz for 30 min, and the stimulation parameters induced visible muscle contractions. The initial EA treatment was performed after completion of the reperfusion and once daily thereafter. The subsequent daily EA treatments were started at the same time points as the beginning of the first EA treatment.

### Lentivirus construction and intracerebroventricular administration

The lentivirus for silencing OTULIN (LV-shOTULIN, 1 × 10^9^ transduction units [TU]/ml) and control lentivirus (LV-Scramble, 1 × 109 transduction units [TU]/ml) were constructed by GenePharma Corporation (Shanghai, China).

Seven days before tMCAO, SD rats received an intracerebroventricular (i.c.v.) injection of LV-shOTULIN or LV-Scramble as described previously (Xu et al. 2018a, b). Briefly, rats were anaesthetized and fixed in a stereotaxic apparatus (Stoelting, USA). LV-shOTULIN or LV-Scramble was injected into the right i.c.v. (bregma coordinates: 1.3 mm lateral, 1.5 mm posterior, and 3.8 mm under the dural surface) using a 10-µl Hamilton syringe (Hamilton Co., Reno, NV, USA) at a rate of 0.5 µl/min, and the needle was left in place for 5 min to prevent backflow. The animals were carefully monitored until recovery from anaesthesia. To verify the effect of gene silencing on OTULIN expression, OTULIN mRNA and protein were detected using RT-qPCR and Western blotting, respectively.

### Neurobehavioural assessment

An independent investigator who was blinded to the experiment performed assessments of neurobehavioural deficits at 72 h using the modified Neurological Severity Score (mNSS) (Chen et al. 2001), rotarod test (Linden et al. 2014) and the inclined board test (Zhang et al. 2006) with some modifications.

The mNSS is a composite test that includes motor (muscle status and abnormal movement), sensory (visual, tactile and proprioceptive), reflex (pinna, corneal and startle), and balance assessments. It is graded on a scale of 0–18, and higher scores indicate more severe injury.

The rotarod test was used to evaluate motor coordination using an accelerating rotarod apparatus (YLS-4C, Shanghai Jinggong Industry Co., Ltd, China). Briefly, each rat was placed on the rotating rod, which accelerated from 4 to 40 rpm per min for three trials with 10-min breaks. A trial ended when the rat fell from the rotating rod or clung to the rod without walking for two consecutive rotations, and the latency to trial ending was recorded. Before tMCAO, all rats were trained to stay on the rotating rod at a speed of 4 rpm three times daily for three days. The pre-tMCAO data were recorded as the internal control. The rotarod test results are presented as the percentages of the mean latency times post-tMCAO compared with pre-tMCAO. Lower scores indicate more severe injury.

An inclined board test was used to assess balance and coordination. Animals were placed on a board (50 × 30 cm) covered with a copper wire mesh (0.2 mm) and stabilized. The board was gradually inclined from the horizontal to the vertical plane. The holding angle at which the animal fell from the board was recorded. The test was repeated and measured three times at two-min intervals. The scores were recorded based on the holding angles: 0, > 70°; 1, 65–69°; 2, 60–64.9°; 3, 55–59.9°; and 4, < 55°. Higher scores indicate more severe injury.

### TTC staining

The cerebral infarct volume was detected using 2,3,5-triphenyltetrazolium chloride (TTC, Sigma-Aldrich, USA) staining as described previously (Xu et al. 2018a, b). Rats were euthanized, and brains were quickly removed and frozen for 20 min at − 20 °C. The brains were coronally sliced into five 2-mm thick sections, stained with 2% TTC at 37 °C for 10 min, then fixed with 10% formaldehyde. Stained sections were photographed using a camera (Canon IXUS, Canon Co., Japan) and analysed in Image-Pro Plus (version 6.0, Media Cybernetics Co., USA). The infarct volume was calculated using the following formula: percentage hemisphere lesion volume = [total infarct volume—(right hemisphere volume—left hemisphere volume)] / left hemisphere volume × 100%.

### Cresyl violet staining

Frozen coronal brain sections were used for cresyl violet staining to label the ischaemic penumbra of the cerebral cortex in rats as previously described (Marchese et al. 2020). The brain sections were dehydrated through descending grades of ethanol (95%, 90%, 80%, 70%, and 50% for 5 min) and rinsed with water. The sections were immersed in a 0.1% cresyl violet solution for 10 min, rinsed quickly in water, dehydrated though ascending grades of ethanol, immersed three times for two min each in xylene, then cover-slipped.

### Nissl staining

Frozen coronal brain sections were dehydrated through descending grades of ethanol (95%, 90%, 80%, 70%, and 50% for 5 min), immersed in a 0.1% cresyl violet solution for 10 min, rinsed quickly in water, rehydrated though ascending grades of ethanol, immersed three times for two min each in xylene, then cover-slipped. The sections were photographed using a digital camera.

### Western blotting

Rats were sacrificed, and the brain tissues in the ischaemic penumbra were rapidly dissected. The tissues were homogenized in RIPA lysis buffer (no. P0013B, Beyotime, Shanghai, China) supplemented with PMSF (Beyotime, Shanghai, China) and phosphatase inhibitors for the detection of phosphorylated proteins. After centrifugation, total protein was extracted from the supernatant. The nuclear and cytoplasmic proteins were extracted using a Nuclear and Cytoplasmic Protein Extraction Kit (no. AR0106, Boster, Beijing, China), and the protein concentrations were detected using a BAC kit (Beyotime, Shanghai, China). SDS-PAGE was used to separate and transfer proteins to PVDF membranes (Millipore Co., USA). After blocking in 5% non-fat milk for two h, the PVDF membranes were incubated with the following primary antibodies at 4 °C overnight: anti-OTULIN polyclonal rabbit antibody (no. 14127, Cell Signaling Technology, USA, 1:1000), anti-NF-κB p65 rabbit antibody (no. 8242, Cell Signaling Technology, USA, 1:1000), anti-IκBα rabbit antibody (no. 4812, Cell Signaling Technology, USA, 1:1000), anti-phospho-IκBα (Ser32) rabbit antibody (no. 2859, Cell Signaling Technology, USA, 1:500), and anti-β-actin rabbit antibody (no. 4970, Cell Signaling Technology, USA, 1:1000). The membranes were incubated with a specific horseradish peroxidase-conjugated secondary antibody for one h at 37 °C. A gel imaging instrument (Vilber Lourmat fusion FX 7 Spectra, France) and analysis software (FUSION-CAPT, France) were used to scan and analyse the immunoblots, respectively.

### Real-time quantitative reverse transcription polymerase chain reaction (RT-qPCR) analysis

Total tissue RNA in the ischaemic penumbra of the cerebral cortex was isolated using TRIzol (Takara Biotechnology, Japan). The mRNA was used as a template to synthesize cDNA using the PrimeScript™ RT reagent kit with gDNA Eraser (TaKaRa) at 42 °C for two min. The synthesized cDNA was used for RT-qPCR in an iQ5 Gradient Real-Time PCR detection system (Bio-Rad Co., USA) with SYBR Green (SYBR Premix Ex Taq™ II, TaKaRa). The following cycling conditions were used: 10 min at 95 °C followed by 40 cycles of 5 s at 95 °C and 30 s at 60 °C. The melting curve was used to analyse the gene specificity of OTULIN and the housekeeping gene β-actin. Relative quantification was performed using the 2^−∆∆Ct^ method. The expression of each targeted gene was normalized to the expression of β-actin in the same sample. All following primer sequences were used in the study: for OTULIN, forward primer: TGTGGCTCCTGAAATGGATATTATG, reverse primer: CTCTGACAGGGATGTTATAGTGCCG; for β-Actin, forward primer: TGTCACCAACTGGGACGATA, reverse primer: GGGGTGTTGAAGGTCTCAAA.

### Immunofluorescence and TUNEL staining

Anaesthetized rats were perfused transcardially with 0.9% saline and 4% formaldehyde, and the brains were removed, fixed in 4% paraformaldehyde for 24 h, and dehydrated with 30% sucrose, 20% sucrose and 15% sucrose. The brains were cut into 10-μm thick coronal sections, incubated with 1% Triton X-100 for 30 min, and blocked with 5% bovine serum albumin (BOSTER Co., USA) for 1 h at 37 °C. The sections were incubated overnight at 4 °C with the following primary antibodies: anti-OTULIN rabbit antibody (bs-14689R, Bioss Co., Beijing, China, 1:50), anti-NeuN mouse antibody (MAB377, Millipore Co., Germany, 1:200), anti-Iba-1 goat antibody (NB100-1028, Novus Co., USA, 1:200), anti-GFAP mouse antibody (A00213, BOSTER Co.), and anti-NF-κB p65 rabbit antibody p65 (#8242, Cell Signaling Technology, USA). The slices were incubated with the following secondary antibodies at 37 °C for one h: Alexa Fluor 594-conjugated goat anti-rabbit IgG (H + L; SA00006-4, Proteintech, 1:200), Alexa Fluor 594-conjugated goat anti-mouse IgG (H + L) (SA00006-3, Proteintech, 1:100), Alexa Fluor 488-conjugated goat anti-mouse IgG (H + L; SA00006-1, Proteintech, 1:200), FITC-conjugated AffiniPure donkey anti-goat IgG (H + L; SA00003-3, Proteintech, 1:200), or 594-conjugated AffiniPure donkey anti-rabbit IgG (H + L; SA00006-8, Proteintech, 1:200). The nuclei were stained with DAPI (Sigma, USA, 1:200). All images were captured using an A1 + R laser confocal microscope (Nikon, Tokyo, Japan). Apoptotic cells were detected using TUNEL assays according to the manufacturer’s instructions (Roche, Basel, Switzerland). The sections were photographed using an A1 + R laser confocal microscope (Nikon, Tokyo, Japan).

Iba-1 is a commonly used microglial marker. The morphology of reactive microglia in the ischaemic penumbra of the cerebral cortex was determined using a classic method (Ito et al. 2001; Sawano et al. 2015). Briefly, Iba-1^+^ cells were categorized into the following three forms according to branch length and thickness and cell body size: (1) ramified microglia, with a small soma, and very fine, long processes; (2) internal microglia, with a larger cell body and thicker processes than ramified microglia; and (3) ameboid microglia, with a large cell body without processes.

The morphological analysis of GFAP^+^ astrocytes was primarily performed using a previously described method (Ramirez-Sanchez et al. 2018). Briefly, GFAP^+^ cells with clearly visible nuclei and soma were selected, and the length of the longest cellular processes (i.e., the distance of the nucleus to the tip of the extension process) was measured to assess the degree of hypertrophy of astrocytes according to a previously described method.

### Immunohistochemistry

Paraffin-embedded brain sections were deparaffinized in xylene, immersed in citrate buffer for antigen retrieval and microwaved for 20 min. Endogenous peroxides were blocked with 3% H_2_O_2_ for 30 min, and nonspecific antigens were blocked with 5% goat serum for 30 min at 37 °C. The sections were incubated at 4 °C overnight with the following primary antibodies: IκBα rabbit monoclonal antibody (ab32518, Abcam, USA, 1:100) and anti-NF-κB p65 rabbit monoclonal antibody (#8242, CST, USA, 1:400). The sections were incubated with secondary horseradish peroxidase-conjugated goat anti-rabbit antibodies (Proteintech™, 1:2000) and stained with 3,3ʹ-diaminobenzidine (DAB) substrate. Images were acquired using an automatic microscope (Olympus, Tokyo, Japan) (Qin et al. 2013).

### Enzyme-linked immunosorbent assay (ELISA)

The levels of tumour necrosis factor alpha (TNF-α), interleukin-1 beta (IL-1β) and interleukin-6 (IL-6) in brain tissues from the ischaemic penumbra of the cerebral cortex were detected using ELISA kits (no. EK0526 96 T, EK0393 and EK0412 96 T, BOSTER Co.) according to the manufacturer’s instructions. The entire experimental flowchart is shown in Fig. 1.

### Statistical analysis

The neurological scores were analysed using Kruskal–Wallis tests followed by post hoc Dunn’s multiple comparison tests. All other data are expressed as the means ± SEM. The expression levels of OTULIN mRNA and protein in the Sham, tMCAO and EA groups were compared using two-way ANOVA with Bonferroni post hoc tests. The percentages of Iba-1^+^OTULIN^+^ cells and NeuN^+^OTULIN^+^ cells in each group were compared using Dunnett’s T3 tests. All other quantitative data were analysed using one-way ANOVA followed by Tukey’s post hoc test for multiple comparisons. Statistical analyses were performed using SPSS 19.0. P < 0.05 was considered statistically significant.

## Results

### EA enhanced OTULIN expression following focal cerebral ischaemia/reperfusion injury

**Fig. 2:**
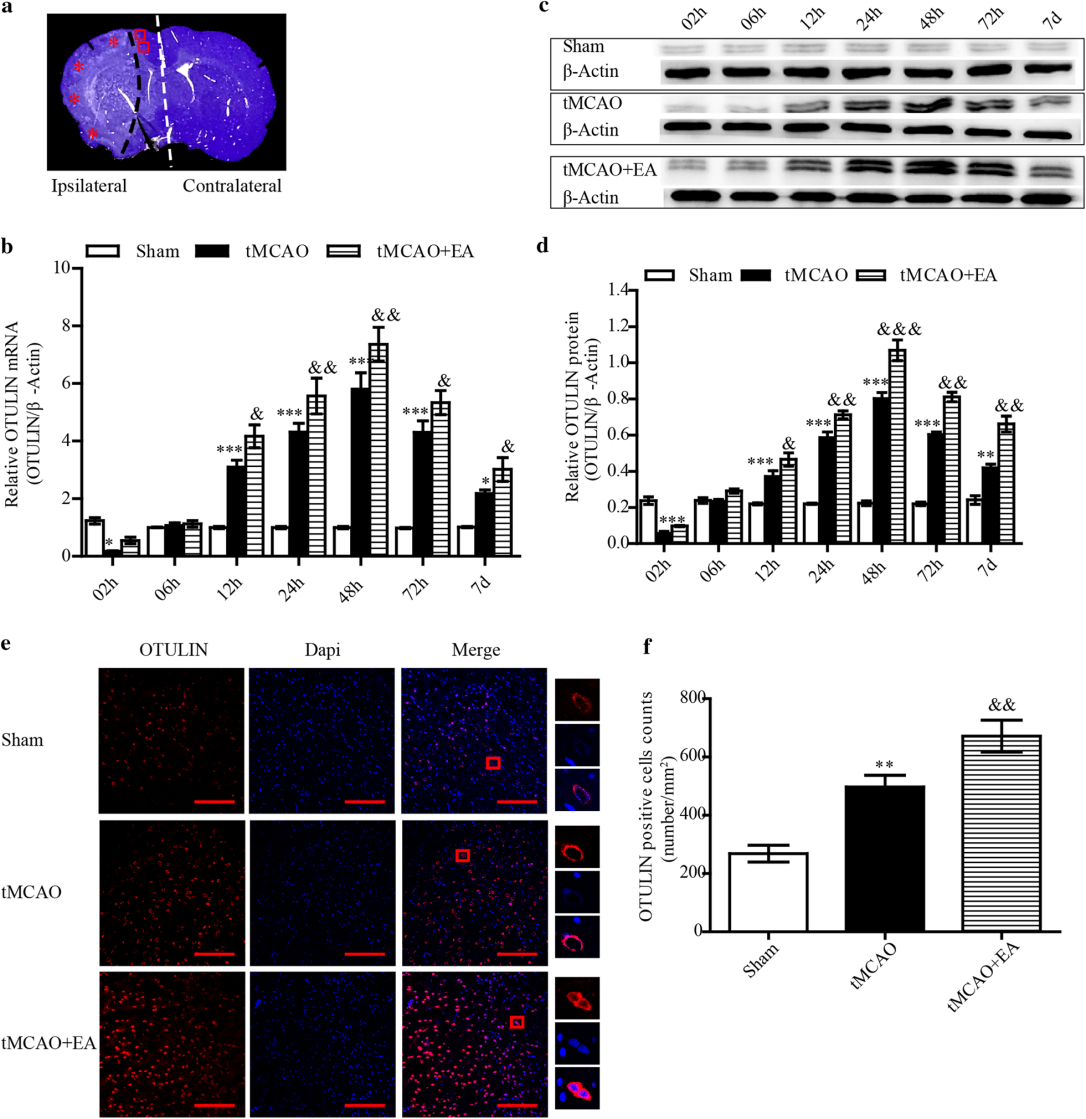
EA enhanced OTULIN expression in ischaemic stroke rats. **a** A coronal brain section was stained with cresyl violet to show the analysed regions (*: ischaemic core area; □: the ischaemic penumbra of the cerebral cortex; the white dotted line separated the ipsilateral and contralateral cerebral hemisphere.). **b** OTULIN mRNA levels at each time point were detected using RT-qPCR (n = 3). **c** OTULIN protein levels in each group were examined using Western blotting. **d** The histogram presents the quantitative analysis of OTULIN protein (n = 3). **e** Immunofluorescence for OTULIN expression in the cortical penumbra was performed 48 h after reperfusion. Scale bar = 100 μm. **f** The histogram presents the quantitative analysis of OTULIN-positive cell counts (n = 8). ***P < 0.001, **P < 0.01, and *P < 0.05 versus the Sham group; &&&P < 0.001, &&P < 0.01, and &P < 0.05 versus the tMCAO group

To detect the effects of EA on the time course of OTULIN expression, OTULIN mRNA and protein in the ischaemic penumbra of the cerebral cortex (Fig. 2a) were measured from 2 h to 7 days after reperfusion using RT-qPCR and Western blotting, respectively. Rats were randomly divided into Sham, tMCAO and tMCAO + EA groups. OTULIN mRNA and protein in the tMCAO group was obviously decreased compared to the Sham group as early as 2 h then increased gradually to peak at 48 h. The concentrations of OTULIN mRNA and protein in the tMCAO + EA group increased significantly compared to the tMCAO group at the indicated time points, except at 2 h and 6 h (Fig. 2b–d).

The immune response of OTULIN protein in the ischaemic penumbra of the cerebral cortex was determined using immunofluorescence at 48 h. The number of OTULIN^+^ cells in the tMCAO group was obviously increased compared to the Sham group (P < 0.01), and the number of cells in the tMCAO + EA group further increased compared to the tMCAO group (P < 0.01). OTULIN protein was primarily located in the cytoplasm, but a small amount of OTULIN was expressed in the nuclei in all groups. (Fig. 2e, f).

### Enhanced OTULIN was primarily expressed in microglia and neurons in focal cerebral ischaemia/reperfusion rats

We examined the main cellular localization of OTULIN protein in the ischaemic penumbra of the cerebral cortex. Rats were randomly divided into Sham, tMCAO and tMCAO + EA groups. OTULIN expression peaked at 24 h, and this time point was selected for observation. Immunofluorescence staining showed that OTULIN co-stained with NeuN, Iba-1 and GFAP.

**Fig. 3:**
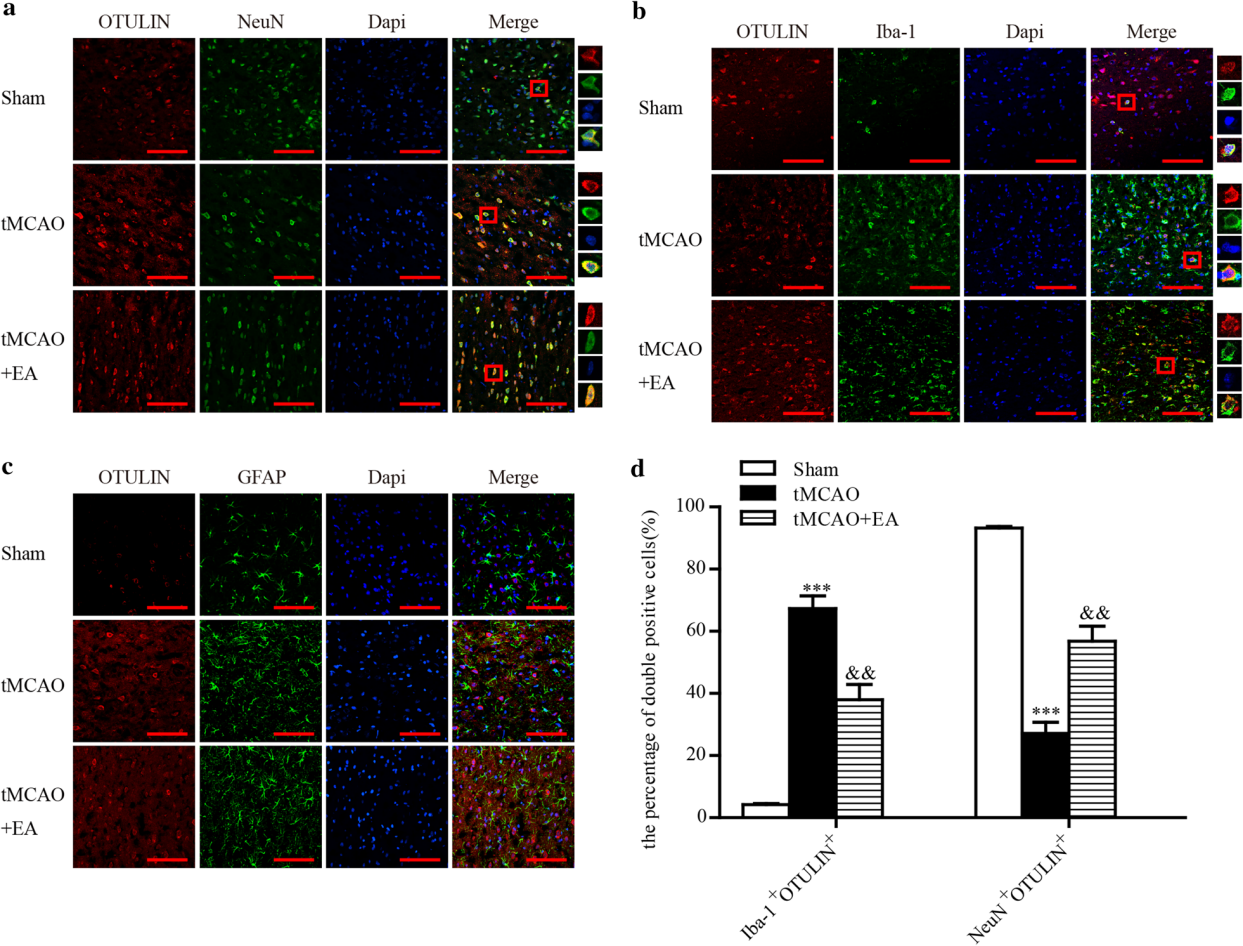
The cellular distribution of OTULIN in ischaemic stroke rats. OTULIN protein (red) co-labelled with neurons (**a** green, identified using NeuN), microglia (**b** green, identified using Iba-1), and astrocytes (**c** green, identified using GFAP). The nuclei were stained with DAPI (blue). **d** The histogram presents the quantitative analysis of the percentages of Iba-1^+^OTULIN^+^ cells and NeuN^+^OTULIN^+^ in each group (n = 8). Scale bar = 100 μm. ***P < 0.001 versus the Sham group; &&P < 0.01 versus the tMCAO group

As shown in Fig. 3, OTULIN was primarily located in NeuN^+^ cells in the Sham group, but a small amount of OTULIN was detected in Iba-1^+^ cells. OTULIN was predominantly expressed in Iba-1^+^ cells in the tMCAO group, and a small amount of OTULIN was observed in a reduced number of NeuN^+^ cells. OTULIN protein was upregulated in NeuN^+^ and Iba-1^+^ cells in the tMACO + EA group compared to the tMCAO group. No OTULIN expression was observed in GFAP^+^ cells in any group. The percentage of Iba-1^+^OTULIN^+^ cells in the tMCAO group was obviously higher than the Sham and tMCAO + EA groups. The Sham group had the highest percentage of NeuN^+^OTULIN^+^ cells. The percentage of NeuN^+^OTULIN^+^ cells in tMCAO + EA group was significantly increased compared to the tMCAO group.

### OTULIN knockdown reversed the neuroprotective effects of EA against ischaemic brain injury

**Fig. 4:**
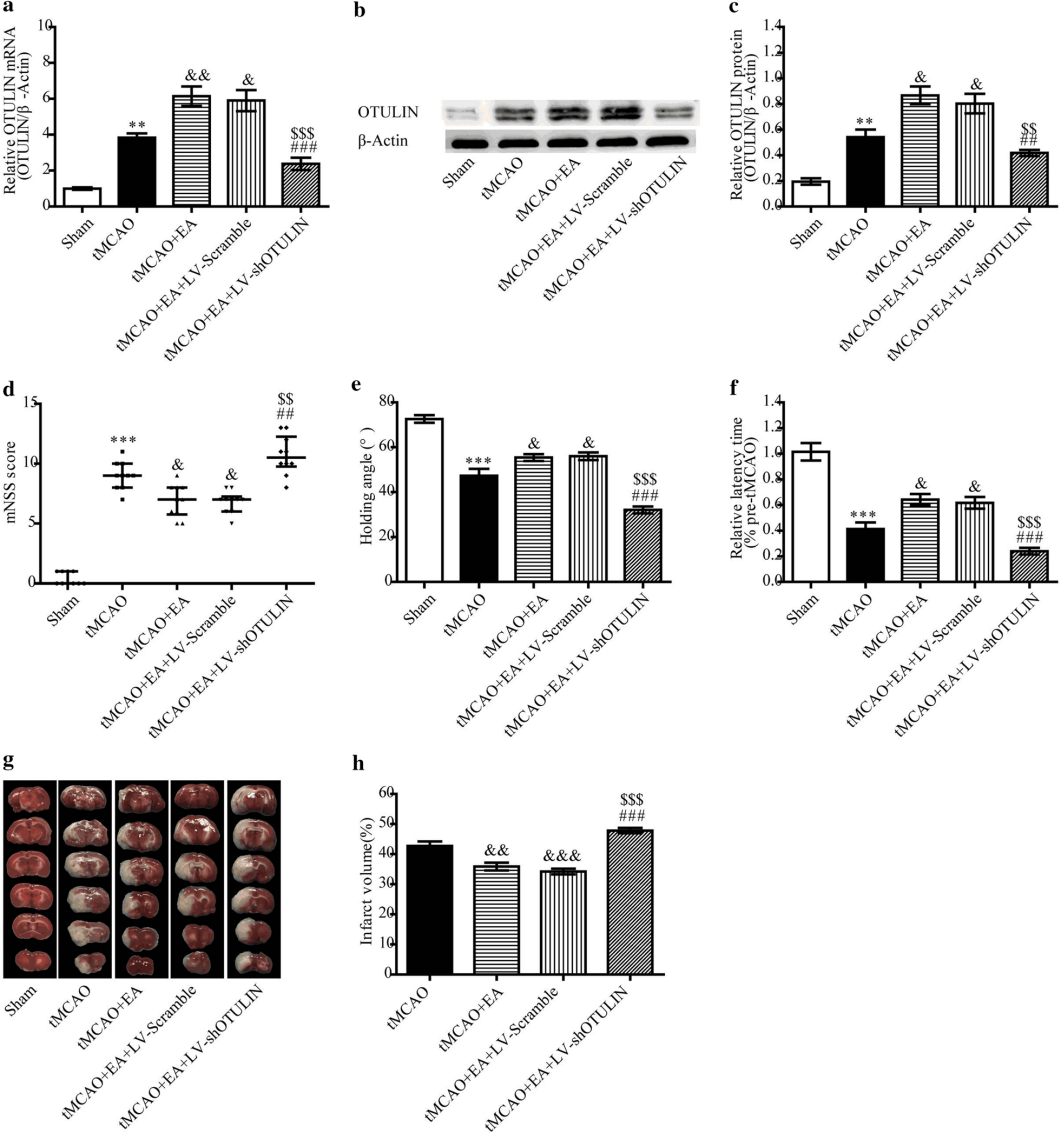
OTULIN silencing inhibited the neuroprotective effect of EA in focal cerebral ischaemia/reperfusion rats. Gene interference with OTULIN expression was assessed using RT-qPCR (**a**, n = 3) and Western blotting (**b** and **c**, n = 3). Neurobehavioural function was evaluated using the mNSS score (**d**, n = 10), inclined board test (**e**, n = 10), and rotarod test (**f**, n = 10). **g** The graph shows an image of TTC (white: infarct area; red: noninfarct area). (**h**, n = 5) The panel presents the quantitative analysis of the cerebral infarct volume. ***P < 0.001 and **P < 0.01 versus the Sham group; &&&P < 0.001, &&P < 0.01, and &P < 0.05 versus the tMCAO group; $$$P < 0.001 and $$P < 0.01 versus the tMCAO + EA group; ###P < 0.001 and ##P < 0.01 versus the tMCAO + EA + LV-Scramble group

To examine whether enhanced OTULIN expression was required for EA to exert its neuroprotective effect, LV-shOTULIN was used to silence OTULIN expression. Rats were randomly divided into five groups: Sham, tMCAO, tMCAO + EA, tMCAO + EA + LV-Scramble, and tMCAO + EA + LV-shOTULIN. Seventy-two hours after reperfusion, the levels of OTULIN mRNA and protein in the tMCAO + EA + LV-shOTULIN group were significantly decreased compared to the tMCAO + EA and tMCAO + EA + LV-scramble groups (Fig. 4a–c).

The neurobehavioural assessment of the mNSS, inclined board test and rotarod test, and cerebral infarct volume detection were performed at 72 h. As shown in Fig. 4d–h, rats in the tMCAO group showed more severe neurobehavioural dysfunction than rats in the Sham group. EA obviously improved neurological function, as demonstrated by a lower mNSS score, higher holding angle and relative latency time than the tMCAO group. However, this improvement was weakened by LV-shOTULIN, which was evidenced by poor neurobehavioural improvement in the tMCAO + EA + LV-shOTULIN group. As shown in Fig. 4g and h, no detectable cerebral infarct volume was found in the Sham group (Fig. 4g, h). The infarct volume in the tMCAO + EA group was decreased compared to the tMCAO group. After OTULIN silencing, EA failed to reduce the infarct volume.

### Enhanced OTULIN expression was necessary for EA to attenuate neuronal injury in focal cerebral ischaemia/reperfusion rats

**Fig. 5:**
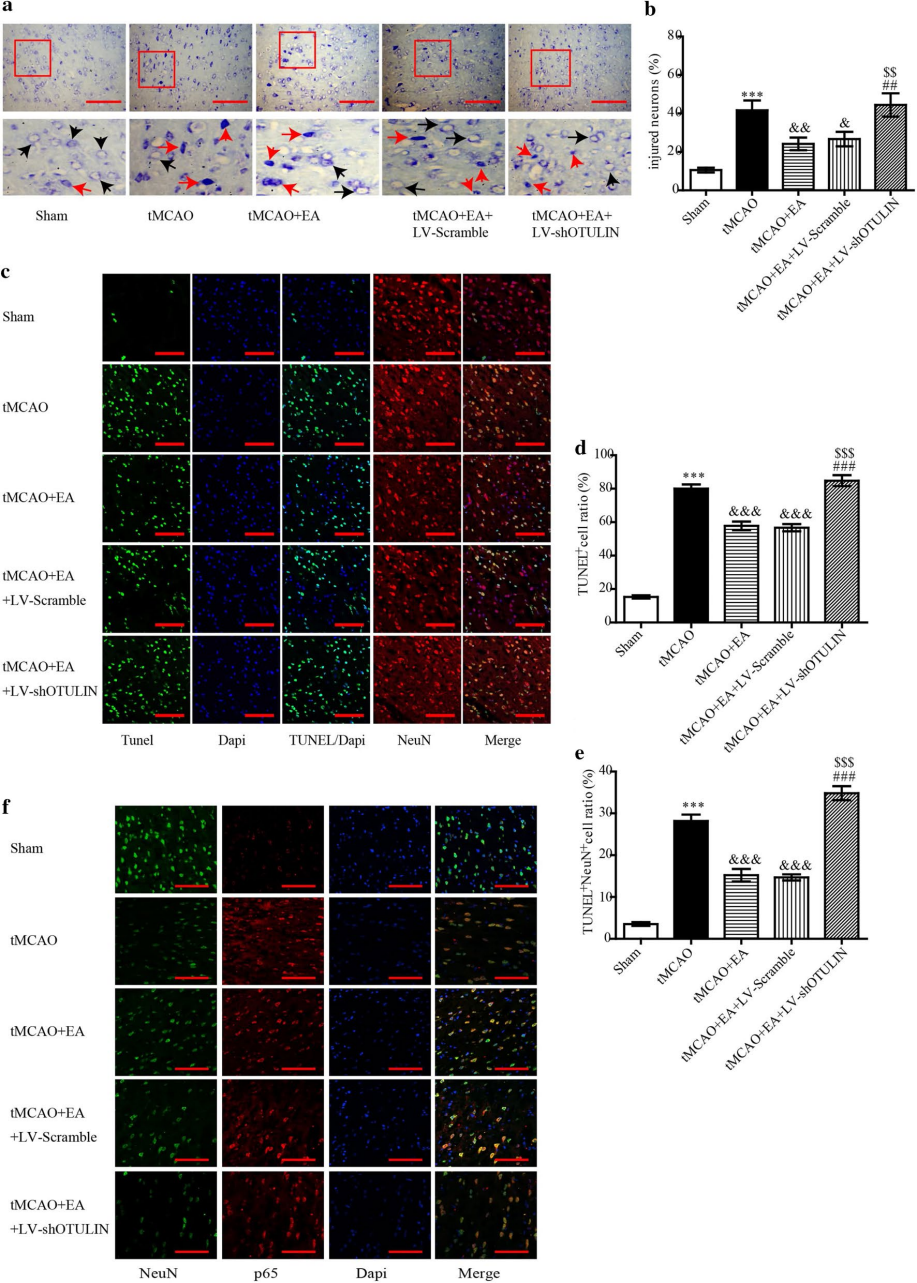
OTULIN was required for EA to attenuate neuronal injury in focal cerebral ischaemia/reperfusion rats. **a** Nissl staining was used to observe the morphology of neurons in the ischaemic penumbra of the cerebral cortex. Black and red arrows represent normal neurons and injured neurons, respectively. (**b**, n = 3) The statistical analysis of injured neurons using Nissl staining. **c** Immunofluorescence was used to detect TUNEL^+^ cells and TUNEL^+^NeuN^+^ cells. Panels **d** (n = 3) and **e** (n = 3) present the quantitative analyses of the TUNEL^+^ and TUNEL^+^NeuN^+^ cells. **f** NeuN and NF-κB p65 were co-stained to observe the nuclear translocation of neuronal NF-κB p65 using immunofluorescence. ***P < 0.001 versus the Sham group; &&&P < 0.001, &&P < 0.01, and &P < 0.05 versus the tMCAO group; $$$P < 0.001 and $$P < 0.01 versus the tMCAO + EA group; ###P < 0.001 and ##P < 0.01 versus the tMCAO + EA + LV-Scramble group. Scale bar = 100 μm

Because OTULIN was also detected in neurons, we investigated the effects of OTULIN in the role of EA attenuation of neuronal injury in the ischaemic penumbra of the cerebral cortex at 24 h. Rats were randomly divided into the following five groups: Sham, tMCAO, tMCAO + EA, tMCAO + EA + LV-Scramble and tMCAO + EA + LV-shOTULIN. Viable neurons were detected using Nissl staining. As shown in Fig. 5a and b, neurons were arranged regularly, and most cells exhibited a normal shape in the Sham group. Most neurons in the tMCAO group were shrunken, deeply stained with a sparsely distributed cytosolic concentration and exhibited karyopyknosis and karyorrhexis. The morphology of neurons in the tMCAO + EA group was improved, with many normal-shaped cells, and the number of viable neurons was increased compared to the tMCAO group (P < 0.01). However, OTULIN silencing obviously blocked this improvement (P < 0.01). Figure 5c–e shows a large number of TUNEL^+^ and TUNEL^+^NeuN^+^ cells in the tMCAO group, and few apoptotic cells were detected in the Sham group. EA treatment significantly decreased the proportion of TUNEL^+^ and TUNEL^+^NeuN^+^ cells compared to the tMCAO group (P < 0.001), and OTULIN silencing inhibited this effect.

We examined whether OTULIN participated in EA regulation of neuronal NF-κB p65 nuclear translocation. As shown in Fig. 5f, neuronal NF-κB p65 was primarily expressed in the nuclei in the tMCAO group. An increased concentration of NF-κB p65 protein was translocated to neuronal cytoplasm in the tMCAO + EA group compared to the tMCAO group, and OTULIN silencing reversed this effect.

### Increased OTULIN expression was required for EA to inhibit the NF-κB signalling pathway in focal cerebral ischaemia/reperfusion rats

**Fig. 6:**
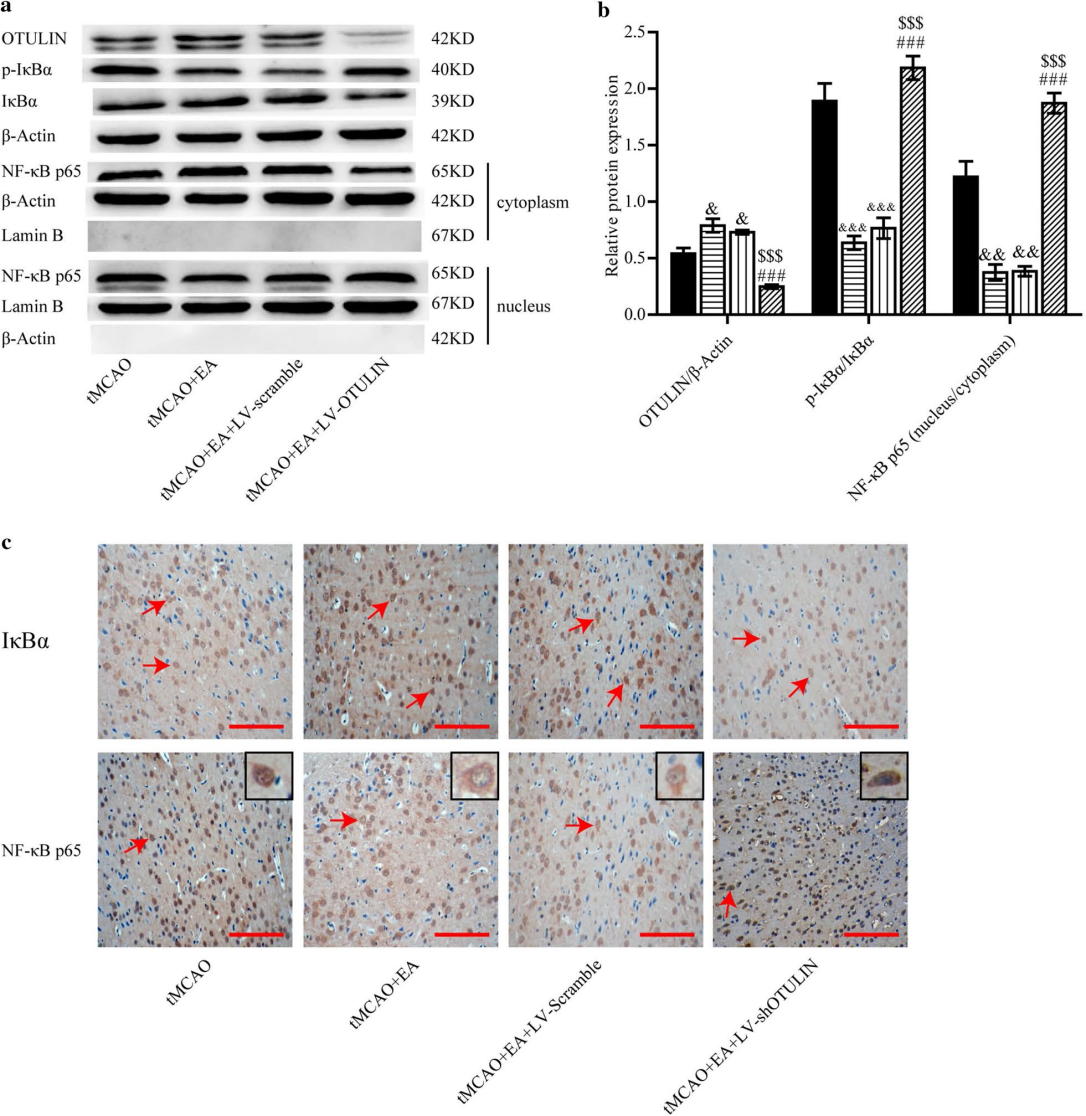
EA inhibited the NF-κB signalling pathway via the upregulation of OTULIN expression. **a** Western blotting was used to detect OTULIN, p-IκBα, IκBα, and cytoplasm/nucleus-p65 proteins. β-Actin served as a loading control for total or cytoplasmic protein, and Lamin B served as a nuclear protein. **b**, n = 3) Quantitative analysis of OTULIN protein, the phosphorylation ratio of IκBα, and nuclear/cytoplasmic NF-κB p65. **c**, n = 6) Immunohistochemistry was performed to detect IκBα and NF-κB p65 proteins. Red arrows indicate IκBα- or NF-κB p65-positive cells. Scale bar = 100 μm. &&&P < 0.001, &&P < 0.01 and &P < 0.05 versus the tMCAO group; $$$P < 0.001 versus the tMCAO + EA group; ###P < 0.001 versus the tMCAO + EA + LV-Scramble group

To further investigate whether enhanced OTULIN was required for EA to inhibit the NF-κB signalling pathway, OTULIN, p-IκBα, IκBα, cytoplasm-p65 and nucleus-p65 proteins from brain tissues in the ischaemic penumbra of the cerebral cortex were detected 24 h after reperfusion. p-IκBα/IκBα represents the phosphorylation ratio of IκBα, and nuclear/cytoplasmic NF-κB p65 represents the nuclear translocation of NF-κB p65 protein. Rats were randomly divided into tMCAO, tMCAO + EA, tMCAO + EA + LV-Scramble and tMCAO + EA + LV-shOTULIN groups. As shown in Fig. 6a and b, increased OTULIN protein content, a lower phosphorylation ratio of IκBα, and attenuated nuclear translocation of p65 protein were detected in the tMCAO + EA group compared to the tMCAO group. Little OTULIN was detected in the MCAO + EA + LV-shOTULIN group, and an elevated phosphorylation ratio of IκBα and increased nuclear translocation of NF-κB p65 were detected compared to the tMCAO + EA and tMCAO + EA + LV-Scramble groups.

Immunohistochemical analysis was also used to detect IκBα and NF-κB p65 proteins 24 h after reperfusion. As shown in Fig. 6c, EA obviously enhanced IκBα staining, and IκBα staining was obviously attenuated in the tMCAO + EA + LV-shOTULIN group compared to the tMCAO + EA group. NF-κB p65 protein was predominantly located in the nucleus in the tMCAO group but partially transferred to the cytoplasm in the tMCAO + EA group. NF-κB p65 staining increased in the nucleus and decreased obviously in the cytoplasm in the tMCAO + EA + LV-shOTULIN group compared to the tMCAO + EA and tMCAO + EA + LV-Scramble groups.

### OTULIN silencing reversed EA suppression of microglial activation after focal cerebral ischaemia/reperfusion injury

**Fig. 7:**
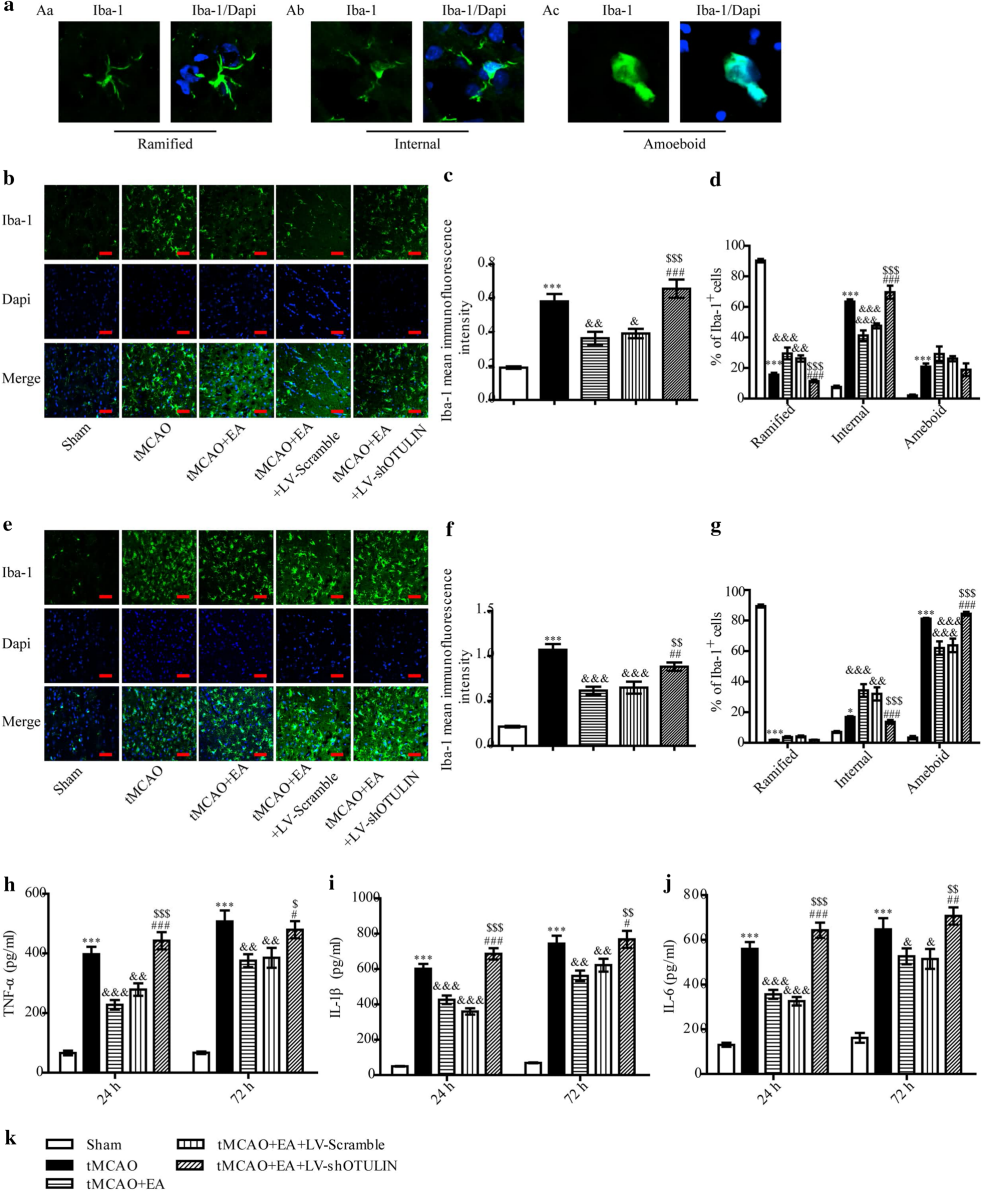
OTULIN silencing weakened the inhibitory effect of EA on microglial activation in focal cerebral ischaemia/reperfusion rats. **A** Representative images of Iba-1^+^ cells in the ischaemic penumbra of the cerebral cortex: **Aa** Ramified cells; **Ab** Internal cells; **Ac** Ameboid cells. The Iba-1^+^ microglial cells in the ischaemic penumbra of cerebral ischaemia were detected using immunofluorescence 24 h (**b**) and 72 h (**e**) after reperfusion. Histograms **c** (n = 6) and **f** (n = 6) present the quantitative analyses of mean Iba-1 immunofluorescence intensity at 24 h and 72 h, respectively. Histograms **d** (n = 6) and **g** (n = 6) present the quantitative analyses of the proportions of ramified, internal, and ameboid cells at 24 h and 72 h, respectively. The contents of TNF-α (**h**, n = 6), IL-1β (**i**, n = 6) and IL-6 (**j**, n = 6) at 24 h and 72 h were detected. Scale bar = 50 μm. ***P < 0.001 and *P < 0.05 versus the Sham group; &&&P < 0.001, &&P < 0.01, and &P < 0.05 versus the tMCAO group; $$$P < 0.001, $$P < 0.01 and $P < 0.05 versus the tMCAO + EA group; ###P < 0.001, ##P < 0.01, and #P < 0.05 versus the tMCAO + EA + LV-Scramble group

The transformation of microglial shape is a manifestation of microglia activation. Microglia located in the ischaemic penumbra of the cerebral cortex primarily exhibited the following three forms: ramified cells (Fig. 7Aa), internal cells (Fig. 7Ab) and ameboid cells (Fig. 7Ac). To investigate the role of OTULIN in EA attenuation of microglial activation, mean Iba-1 immunofluorescence intensity and the proportions of microglia with different forms were analysed quantitatively. All rats were randomly divided into the following five groups: Sham, tMCAO, tMCAO + EA, tMCAO + EA + LV-Scramble and tMCAO + EA + LV-shOTULIN.

The mean Iba-1 immunofluorescence intensity in each group was analysed. Twenty-four and 72 h after reperfusion, the mean Iba-1 immunofluorescence intensity was very low in the Sham group and increased sharply in the tMCAO group, but it was obviously reduced in the tMCAO + EA group, as shown in Fig. 7. After OTULIN silencing, the mean Iba-1 immunofluorescence intensity in the tMCAO + EA + LV-shOTULIN group was increased significantly compared to the tMCAO + EA and tMCAO + EA + LV-Scramble groups.

The effects of EA and OTULIN silencing on microglia shape in the ischaemic penumbra were analysed. As shown in Fig. 7b, d, microglia in the tMCAO group primarily exhibited an internal shape at 24 h. The proportion of internal microglia was decreased in the tMCAO + EA group, and the proportion of ramified microglia obviously increased compared to the tMCAO group. After OTULIN silencing, the proportion of ramified microglia was significantly lower in the tMCAO + EA + LV-shOTULIN group than the tMCAO + EA and tMCAO + EA + LV-Scramble groups, and the proportion of internal microglia was significantly higher. At 72 h, activated microglia primarily exhibited an ameboid form (Fig. 7e, g). The proportion of internal microglia was increased in the tMCAO + EA group compared to the tMCAO group, and the proportion of ameboid microglia was obviously decreased. The proportion of internal microglia was obviously decreased in the tMCAO + EA + LV-shOTULIN group compared to the tMCAO + EA and tMCAO + EA + LV-Scramble groups, and the proportion of ramified microglia was higher.

To investigate the effect of OTULIN silencing on EA suppression of pro-inflammatory cytokine secretion, the contents of TNF-α, IL-1β and IL-6 were detected at 24 h and 72 h using ELISA. As shown in Fig. 7h–j, the levels of TNF-α, IL-1β, and IL-6 in the tMCAO group were significantly higher than the Sham group, and the levels decreased after EA treatment. After the silencing of OTULIN expression, the contents of TNF-α, IL-1β and IL-6 in the tMCAO + EA + LV-shOTULIN group were significantly higher than the tMCAO + EA and tMCAO + EA + LV-Scramble groups.

### OTULIN reversed the suppression of astrogliosis by EA in focal cerebral ischaemia/reperfusion rats

Although OTULIN protein was not detected in astrocytes, we examined whether the attenuated neuroinflammation adversely affected the activation of astrocytes. Rats were randomly divided into Sham, tMCAO, tMCAO + EA, tMCAO + EA + LV-Scramble and tMCAO + EA + LV-shOTULIN groups. Immunofluorescence was used to observe GFAP^+^ astrocytes in the ischaemic penumbra of the cerebral cortex at 24 h and 72 h. The mean immunofluorescence intensity of GFAP and the mean longest process of GFAP^+^ cells were quantitatively analysed.

**Fig. 8:**
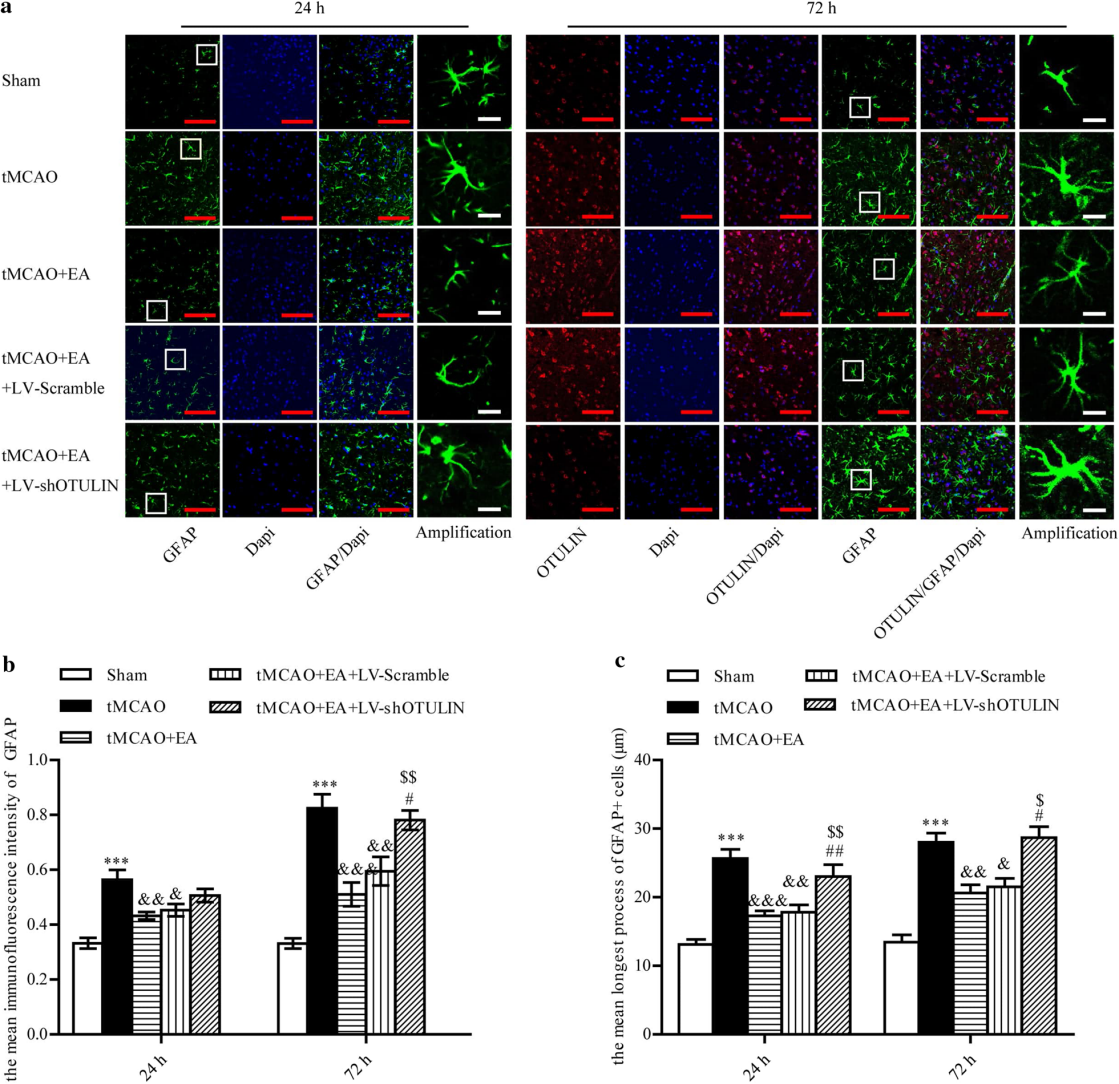
OTULIN was involved in EA attenuation of astrogliosis in focal cerebral ischaemia/reperfusion rats. **a** Immunofluorescence was used to stain astrocytes at 24 h and 72 h. (**b**, n = 6) The mean immunofluorescence intensity of GFAP at 24 h and 72 h was analysed quantitatively. (**c**, n = 6) The mean longest process of GFAP^+^ cells was quantitatively analysed. ***P < 0.001 versus the Sham group; &&&P < 0.001, &&P < 0.01, &P < 0.05 versus the tMCAO group; $$P < 0.01 and $P < 0.05 versus the tMCAO + EA group; #P < 0.05 versus the tMCAO + EA + LV-Scramble group. Scale bar (red) = 100 μm, Scale bar (white) = 10 μm

The mean immunofluorescence intensity of GFAP and the mean longest process of GFAP^+^ cells increased significantly in the tMCAO group compared to the Sham and EA group at 24 h and 72 h. After OTULIN silencing, the mean immunofluorescence intensity of GFAP in the tMCAO + EA + LV-shOTULIN group was significantly higher than the tMCAO + EA group at 72 h, but not at 24 h, and the mean longest process of GFAP^+^ cells was increased significantly compared to the tMCAO + EA group and tMCAO + EA + LV-Scramble group at 24 h and 72 h (Fig. 8).

## Discussion

OTULIN is a crucial negative regulator of the NF-κB signalling pathway, and it is critical in the prevention of autoinflammatory diseases (Damgaard et al. 2016, 2020; Zhou et al. 2016) and protection of embryonic lethality (Rivkin et al. 2013). Genetic mutation of OTULIN in humans causes a severe inflammatory phenotype, and patients exhibit amplified activation of the NF-κB pathway (Damgaard et al. 2016). Mice that lacked OTULIN in myeloid cells exhibited enlarged lymphoid organs and liver, increased immune cell infiltration in the liver, and increased cytokine levels in serum (Damgaard et al. 2016). We previously demonstrated that OTULIN overexpression ameliorated microglial activation and neuroinflammation via suppression of the NF-κB pathway in cerebral ischaemia/reperfusion rats (Xu et al. 2018a, b). Rapidly decreased OTULIN was detected as early as 2 h following perfusion in the present study. We hypothesise that hyperacute brain injury inhibits brain cells from synthesizing OTULIN. Endogenous OTULIN expression subsequently increased gradually with a peak at 48 h to exert endogenous protection. We previously demonstrated that enhanced OTULIN exerted a neuroprotective role in ischaemic stroke.

EA treatment effectively improves ischaemic brain injury. EA treatment at particular acupoints activates afferent fibres that send signals to the spinal cord (Zhao 2008). The most frequently used acupoints in basic studies include Baihui (GV20), Zusanli (ST36), Quchi (LI11), Shuigou (GV26), Dazhui (GV14), and Hegu (LI4) (Chavez et al. 2017). However, the stimulation of different acupuncture points with various frequencies and intensities may exert different effects following cerebral ischaemia. Wang et al. (2021) found that EA treatment at the Baihui (GV20) and Shenting (GV24) acupoints with a 2-mA intensity and 1–20 Hz (adjusted to the muscle twitch threshold) improved learning and memory functions in ischaemic stroke rats. Xu H et al. (Xu et al. 2014) discovered that EA at Baihui (DU20) and left Zusanli (ST36) in rats with cerebral ischemia/reperfusion injury at a frequency of 2 Hz with an intensity of 1 mA for 20 min once daily decreased the expression of HSP90 and pro-inflammatory mediators. EA treatment at different acupoints, frequencies and intensities may regulate neuroinflammation following cerebral ischemia via different signalling pathways (Long et al. 2019; Ma et al. 2019; Geng et al. 2020). However, the exact mechanisms underlying the beneficial anti-inflammatory effects of EA in the treatment of stroke are not clear. We demonstrated that EA at "Baihui (GV 20)," "Hegu (L14)," and "Taichong (Liv3)" acupoints at an intensity of 1 mA and a frequency of 2/20 Hz alleviated brain injury and reduced the neuroinflammatory response via inhibition of the NF-κB signalling pathway following cerebral ischaemia (Qin et al. 2013; Xie et al. 2016; Zhan et al. 2016; Jiang et al. 2017; Xu et al. 2018a, b). The present research showed, for the first time, that OTULIN was located primarily in neurons and microglia in a normal physiological state and following ischaemic stroke, but no OTULIN protein was detected in astrocytes. EA treatment promotes OTULIN gene transcription and protein synthesis in the brain, and enhanced OTULIN expression was primarily located in neurons. Notably, the silencing of OTULIN obviously reversed the neuroprotective effects of EA, which indicated that OTULIN was involved in the anti-preventive role of EA following acute ischemic stroke.

The NF-κB signalling pathway is widely activated in brain cells after cerebral ischaemia, but its role in neuronal injury is controversial. Some studies reported that neuronal conditional NF-κB knockout mice exhibited impaired synaptic transmission, spatial memory formation, and plasticity (O'Mahony et al. 2006). Other studies showed that NF-κB activation in neurons contributed to neuronal cell death in cerebral ischaemia (Zhang et al. 2005; Ridder and Schwaninger 2009; Ingrassia et al. 2012). Sarnico et al. (2009) found that the overexpression of p65 reduced neuron susceptibility to anoxia, and p65 silencing significantly reduced neuronal cell death in vitro. Although the functional consequences of neuronal NF-κB and neuronal survival are disputed, EA decreased neuronal apoptosis and improved neurological function via the inhibition of NF-κB activation in the whole brain. The present study showed that EA alleviation of neuronal injury and apoptosis was accompanied by enhanced OTULIN expression and suppressed activation of the neuronal NF-κB signalling pathway, and these findings were obviously reversed when OTULIN expression was inhibited. However, whether enhanced neuronal OTULIN is directly required for the attenuating effects of EA on neuronal injury is not clear and needs further investigation because this effect may be partially attributed to the alleviated neuroinflammation.

EA treatment reduces microglial activation after cerebral ischaemia/reperfusion injury, and the degree of microglial activation is often assessed by calculating the number of Iba-1^+^ cells or mean Iba-1 immunofluorescence intensity (Han et al. 2015; Liu et al. 2016; Zhan et al. 2016; Jiang et al. 2017; Xu et al. 2018a, b). EA also enhanced microglial OTULIN expression in the present study, and OTULIN silencing effectively reversed the inhibitory effect of EA on microglial activation, as shown by the increased mean Iba-1 immunofluorescence intensity, which strongly indicates that enhanced OTULIN expression is necessary for EA to alleviate microglial activation.

Morphological changes in microglia are a reflection of microglial activation (Soltys et al. 2005; Heindl et al. 2018; Li et al. 2018). However, little was known about the effect of EA on microglial morphology following cerebral ischaemia. We quantitatively analysed microglia with different shapes using immunofluorescence and revealed that EA inhibited the transformation of microglia from the ramified to internal state at 24 h and the transformation of microglia from the internal to ameboid shape at 72 h. Notably, OTULIN silencing partially inhibited these transformations, which suggests that OTULIN is involved in EA inhibition of the transformation of microglia from a resting to an active state. Consistent with our hypothesis, OTULIN silencing reversed the inhibitory effect of EA on the secretion of pro-inflammatory factors in the ischaemic cerebral cortex. EA effectively inhibited the activation of microglia and the secretion of inflammatory mediators via regulation of OTULIN expression.

Astrocytes are the most abundant cells in the CNS and change to a reactive phenotype (so-called reactive astrogliosis) that is characterized by enhanced glial fibrillary acidic protein (GFAP) expression and cellular hypertrophy (Sofroniew 2009; Pekny and Pekna 2014; Rossi 2015) in response to ischaemic stroke. Numerous studies demonstrated that EA inhibited astrocyte activation induced by acute cerebral ischaemia/reperfusion injury (Tian et al. 2016; Zhan et al. 2016; Kim et al. 2018), traumatic brain injury (Tang et al. 2016), neuropathic pain (Liang et al. 2016), and inflammatory pain (Liao et al. 2017). Although astrogliosis plays a dual role in ischaemic stroke, the inhibition of excessive astrogliosis is beneficial (Zhang et al. 2018a) because reactive astrogliosis and glial scar formation primarily underlie functional recovery difficulty after cerebral ischaemia (Cregg et al. 2014; Cheon et al. 2016; Liu and Chopp 2016). EA inhibits astrogliosis (Tian et al. 2016; Zhan et al. 2016), which is beneficial to the anti-inflammatory effect of EA (Zhan et al. 2016). Microglia and astrocytes are the primary immune cell types that quickly respond to cerebral ischaemia, but their functions are increasingly recognized as complex, interactive sometimes synergistic (Liddelow et al. 2017; Zhang et al. 2018b). The complex cell–cell interactions have hampered the mechanistic understanding of astrocyte reactivity. OTULIN was required for EA to alleviate microglial activation and neuroinflammation, and we examined whether astrogliosis was affected after silencing OTULIN. Although no OTULIN was detected in astrocytes, EA failed to suppress astrocyte activation after OTULIN silencing. This result may be related to the attenuated microglia activation because reactive microglia are required to induce reactive astrocytes via the secretion of various inflammatory factors, such as TNF-α, IL-1α, and complement component 1q (C1q) (Liddelow et al. 2017). However, the specific mechanisms need deeper study.

There are some limitations in this study. OTULIN is a new type of deubiquitinating enzyme, and how the EA-induced up-regulated OTULIN inhibited activation of the NF-κB signalling pathway is not known. IKKγ is an important modification target of OTULIN in the NF-κB signalling pathway, and the mechanisms of changes in its ubiquitination modification level are not known. These mechanisms will be elucidated in subsequent studies.

## Conclusion

Our data highlight the temporal and spatial distribution of OTULIN in the cerebral cortex following ischaemic stroke and revealed that enhanced OTULIN was required for EA to alleviate neuronal injury and activate glial cells, which was associated with the NF-κB signalling pathway. Although there are still many unknown mechanisms, our results support OTULIN as a new target that exerts a neuroprotective role in ischaemic stroke. However, this role may involve a complex process that requires the participation and coordination of a variety of cells.

## Data Availability

The datasets used or analysed in the current study are available from the corresponding author on reasonable request.

## Abbreviations

tMCAO: Transient middle cerebral artery occlusion
OTULIN: Ovarian tumor domain deubiquitinase with linear linkage specificity
EA: Electroacupuncture
CCA: Common carotid artery
MCA: Middle cerebral artery
CNS: Central nervous system
i.c.v.: Intracerebroventricular
TTC: 2,3,5-Triphenyltetrazolium chloride
Iba-1: Ionized calcium binding adapter molecule 1
IκBα: NF-κB inhibitor protein alpha
NF-κB: Nuclear transcription factor kappa B
TNF-α: Tumour necrosis factor alpha
IL-1β: Interleukin-1 beta
IL-6: Interleukin-6
RT-qPCR: Real-time quantitative polymerase chain reaction
ELISA: Enzyme-linked immunosorbent assay
